# Melhorando os Resultados de Pacientes com Doença Coronariana Aguda na Vida Real: O Caso de Aplicar na Prática o que já Sabemos dos Estudos Clínicos

**DOI:** 10.36660/abc.20230283

**Published:** 2023-06-07

**Authors:** Stefano Garzon, Pedro A. Lemos

**Affiliations:** 1 Hospital Israelita Albert Einstein São Paulo SP Brasil Hospital Israelita Albert Einstein , São Paulo , SP – Brasil; 2 Instituto do Coração HCFMUSP São Paulo SP Brasil Instituto do Coração – InCor, HCFMUSP , São Paulo , SP – Brasil

**Keywords:** Doenças Cardiovasculares/mortalidade, Doença Arterial Coronariana, Fatores de Risco, Países em Desenvolvimento, Pobreza, Intervenção Coronária Percutânea, Sistema Único de Saúde

As doenças cardiovasculares são uma das principais causas de morte no mundo, tanto em países desenvolvidos quanto em países em desenvolvimento, ^1^ e, de acordo com a Organização Mundial da Saúde, espera-se que 75% dessas mortes ocorram em países em desenvolvimento. ^2^ A privação socioeconômica é um dos principais determinantes de doenças cardiovasculares mortalidade associada a maior exposição a fatores de risco e mortes prematuras. ^3^ As doenças cardiovasculares também têm um grande impacto na economia. Um estudo recente realizado na Austrália ^4^ projeta os custos totais de saúde das doenças cardiovasculares de 2020 a 2029 em quase 62 bilhões de dólares australianos, com perdas de produtividade de quase 79 bilhões, com um custo total superior a 140 bilhões em 10 anos.

O Brasil é um país em desenvolvimento de dimensões continentais, com marcadas disparidades regionais, raciais e socioeconômicas. ^5^ A partir da década de 1960, o país passou por uma rápida mudança demográfica, com uma população envelhecendo rapidamente e passando de ser predominantemente rural para urbana. Grande parte da população brasileira está exposta a fatores de risco conhecidos para doenças cardiovasculares, como baixa escolaridade, maus hábitos de vida e alimentação, tabagismo, obesidade, poluição do ar e fatores de risco mal controlados, como hipertensão, diabetes e hiperlipidemia, todas associadas a maior mortalidade cardiovascular. ^6^ Embora o Brasil tenha um Sistema Único de Saúde (SUS), o acesso à saúde é altamente heterogêneo, superlotado e subfinanciado, e frequentemente não oferece atendimento adequado à população brasileira. ^7^

O artigo de Bruno et al. ^8^ relata os resultados de um registro prospectivo de síndromes coronarianas agudas atendidas no Hospital Universitário, hospital secundário da Universidade de São Paulo. No estudo, entre 800 pacientes tratados, o tratamento com intervenção coronária percutânea (ICP) foi associado a uma menor probabilidade de morte por todas as causas (HR 0,42), doença cardiovascular (HR 0,39) ou doença arterial coronariana (HR 0,24). Notavelmente, os benefícios de sobrevida da ICP foram mantidos, mesmo em um ambiente onde a angiografia coronária invasiva levou em média 4 dias para ser realizada, já que o hospital não possui um laboratório de cateterismo.

Em uma revisão recente, Bhatt, Lopes e Harrington destacam a importância de uma estratégia invasiva precoce ou terapia fibrinolítica quando o acesso imediato à ICP não está disponível para reduzir a mortalidade em pacientes com IAMCSST, bem como a angiografia coronária invasiva imediata para pacientes com IAMSSST de alto risco. ^9^ Essas também são as recomendações das diretrizes mais recentes, pois unanimemente enfatizam a importância da estratificação invasiva oportuna. ^10 - 13^

Como visto na matéria, mesmo em um hospital universitário da cidade mais rica do país, os pacientes ainda esperam dias por uma coronariografia invasiva. Infelizmente, isso ainda reflete como funciona o sistema público de saúde brasileiro. A realidade da maioria das regiões do Brasil, e até mesmo da cidade de São Paulo, provavelmente é diferente (para pior). As desigualdades regionais são difíceis de avaliar, mas certamente esse é um cenário um tanto privilegiado, pois muitas vezes o acesso até mesmo à atenção básica adequada é difícil. Embora as mortes cardiovasculares venham diminuindo no Brasil ^5^ e haja indícios de melhora geral em nossa população, ^14^ podemos perceber no presente artigo que ainda estamos longe de oferecer assistência médica adequada à nossa população. Políticos e formuladores de políticas devem ter em mente que investir em saúde é bom para a saúde de sua população e também para a economia, pois o investimento em prevenção e tratamento e o crescimento econômico andam de mãos dadas. ^15^
