# Supplementary material for: Melhorando os Resultados de Pacientes com Doença Coronariana Aguda na Vida Real: O Caso de Aplicar na Prática o que já Sabemos dos Estudos Clínicos
Source: Arq Bras Cardiol. 2023 Jun 7;120(5):e20230283. [Article in Portuguese] doi: 10.36660/abc.20230283 (PMC12092029; doi:10.36660/abc.20230283)
Supplement: Translation

# Translation: Improving Outcomes of Patients with Acute Coronary Disease in Real Life: The Case of Applying in Practice What We Already Know from Clinical Studies

**Authors:** Stefano Garzon, Pedro A. Lemos

**Affiliations:** 1 Brasil Hospital Israelita Albert Einstein, São Paulo, SP – Brasil; 2 Brasil Instituto do Coração – InCor, HCFMUSP, São Paulo, SP – Brasil

**Keywords:** Cardiovascular Diseases/mortality, Coronary Artery Disease, Risk Factors, Development Countries, Poverty, Percutaneous Coronary Intervention, Unified System Health

Cardiovascular diseases are one the leading causes of death in the world in both developed and developing countries,^1^and according to the World Health Organization, 75% of these deaths are expected to happen in developing countries.^2^Socioeconomic deprivation is a major determinant of cardiovascular mortality associated with more exposure to risk factors and premature deaths.^3^Cardiovascular diseases also take a great toll on the economy. A recent study conducted in Australia^4^projects a total healthcare cost of cardiovascular diseases from 2020 to 2029 of almost 62 billion Australian dollars, with productivity losses of almost 79 billion, with a total cost surpassing 140 billion over 10 years.

Brazil is a developing country of continental dimensions, with marked regional, racial, and socioeconomic disparities.^5^From the 1960s onwards, the country underwent a fast demographic change, with a rapidly aging population and transitioning from predominantly rural to urban. A large proportion of the Brazilian population is exposed to known risk factors of cardiovascular disease, such as low educational levels, poor living and dietary habits, tobacco use, obesity, air pollution, and poorly-controlled risk factors such as hypertension, diabetes, and hyperlipidemia, all of which are associated with higher cardiovascular mortality.^6^Although Brazil has a Universal Healthcare System (SUS), healthcare access is highly heterogeneous, overcrowded, and underfunded, and frequently fails to provide proper care to the Brazilian population.^7^

The article by Brunto et al.^8^reports the results of a prospective registry of acute coronary syndromes treated at the Hospital Universitário, a secondary-care hospital of the University of São Paulo. In the study, among 800 treated patients, treatment with percutaneous coronary intervention (PCI) was associated with a lower probability of death by all causes (HR 0.42), cardiovascular disease (HR 0.39), or coronary artery disease (HR 0.24). Notably, PCI’s survival benefits were maintained, even in a setting where invasive coronary angiography took a median of 4 days to be performed, as the hospital does not have a catheterization laboratory.

In a recent review, Bhatt, Lopes, and Harrington underline the importance of an early invasive strategy or fibrinolytic therapy when immediate access to PCI is not available for reducing mortality in STEMI patients, as well as immediate invasive coronary angiography for high-risk NSTEMI patients.^9^These are also the recommendations of the most recent guidelines, as they unanimously stress the importance of timely invasive stratification.^10 - 13^

As seen in the article, even at a university hospital in the wealthiest city in the country, patients still wait days for invasive coronary angiography. Sadly, this still reflects how the Brazilian public health system works. The reality of most regions in Brazil, and even in the city of São Paulo, is probably different (for the worst). Regional inequalities are difficult to assess, but this is certainly a somewhat privileged scenario, as access to even proper primary care is often difficult. Although cardiovascular deaths have been declining in Brazil^5^and there are indications of overall improvement in our population,^14^we can see from the present article that we are still far from offering adequate healthcare to our population. Politicians and policymakers should keep in mind that investing in healthcare is good for the health of your population and the economy, as an investment in prevention and treatment and economic growth walk hand-in-hand.^15^
